# Increased Levels of Phosphorylated-P38α Induce WNT/β-Catenin and NGF/P75NTR/TrkA Pathways Disruption and SN56 Cell Death following Single and Repeated Chlorpyrifos Treatment

**DOI:** 10.3390/foods13152427

**Published:** 2024-08-01

**Authors:** Paula Moyano, Andrea Flores, María de la Cabeza Fernández, Jimena García, Javier Sanjuan, José Carlos Plaza, Javier Del Pino

**Affiliations:** 1 Pharmacology and Toxicology Department, Veterinary School, Complutense University, 28040 Madrid, Spainjdelpino@pdi.ucm.es (J.D.P.); 2 Chemistry in Chemical Sciences Department, Pharmacy School, Complutense University, 28040 Madrid, Spain; mafern60@ucm.es; 3 Legal Medicine, Psychiatry and Pathology Department, Medicine School, Complutense University, 28040 Madrid, Spain

**Keywords:** chlorpyrifos, basal forebrain cholinergic neurons, P38α, WNT/β-Catenin pathway, NGF/P75^NTR^/TrkA pathway

## Abstract

Chlorpyrifos (CPF) biocide, exposure to which is mainly produced in the human population through diet, induces several neurotoxic effects. CPF single and repeated exposure induces memory and learning disorders, although the mechanisms that produce these outcomes are complex and not well understood. CPF treatment (single and repeated) of cholinergic septal SN56 cells induced an increase in phosphorylated-P38α levels that led to WNT/β-Catenin and NGF/P75^NTR^/TrkA pathways disruption and cell death. These results provide new knowledge on the mechanisms that mediate CPF basal forebrain cholinergic neuronal loss induced by CPF single and repeated exposure and can help unravel the way through which this compound produces cognitive decline and develop efficient treatments against these effects.

## 1. Introduction

Chlorpyrifos (CPF), an organophosphate biocide, is widely used in agricultural application and has been reported to be detected in several food products, which is a cause of concern for human health, especially after chronic exposure through diet [1]. CPF induces many neurotoxic effects such as learning and memory disruption in rats after single and repeated treatment [2,3,4]. CPF has also been associated with cognitive decline in epidemiological studies on product applicators [2,4]. CPF mechanisms that mediate these effects are complex and not all of them are known.

Acute and repeated CPF treatment produces basal forebrain (BF) cholinergic neuronal death [5], which has been associated with learning and memory dysfunction [6]. Therefore, the cognitive decline induced by CPF could be mediated through BF cholinergic neurodegeneration [5,7]. CPF alters the β-Catenin pathway in MCF-7 and MBA-MD-231 human breast cancer cells [8]. The WNT/β-Catenin pathway has been associated with cognitive function regulation and cell viability maintenance, leading to cognitive decline and cell death after its repression [9,10]. CPF was also shown to produce p75 neurotrophin receptor (P75^NTR^) overexpression in BF cholinergic neurons (BFCNs), mediating, in part, the cell death observed [7]. However, no studies have been developed to determine the involvement of the nerve growth factor (NGF)/P75^NTR^/tropomyosin-receptor-kinase-A (TrkA) pathway in this effect. NGF gene expression produces a preproNGF protein, which is degraded to proNGF and finally to mature NGF (mNGF) through tissue plasminogen activator (t-PA) and other proconvertases action [11]. mNGF mediates the maintenance of BFCN viability and cognition through TrkA activation [11], but proNGF activation of the P75^NTR^ receptor induces cell death [11,12]. The final effect on cell viability depends on mNGF/proNGF proteins and P75^NTR^/TrkA receptors ratios, leading to one of these receptors being mainly activated [11,12,13]. Otherwise, CPF was shown to increase phosphorylated-P38α levels, leading to human neuroblastoma SH-SY5Y cell death [14]. P38α has been reported to mediate WNT/β-Catenin and NGF/P75^NTR^/TrkA pathways disruption, selective BFCN death, and cognitive decline [15,16,17,18].

Accordingly, we hypothesized that CPF could increase phosphorylated-P38α levels, decreasing WNT/β-Catenin and NGF/P75^NTR^/TrkA pathways activation, leading to BF cholinergic SN56 cell death. To test this hypothesis, *P75^NTR^*- and *P38α*-silenced or wild-type BF cholinergic SN56 cells were treated singly and repeatedly with CPF (0.1 µM to 50 µM; Sigma, Madrid, Spain) either alone or in combination with recombinant NGF (rNGF; MyBioSource, San Diego, CA, USA) and β-Catenin (rβ-Catenin; MyBioSource, San Diego, CA, USA) proteins.

## 2. Materials and Methods

### 2.1. Culture Procedure

CPF has been shown, in many research articles, to be converted into CPF-oxon (CPFO), which is quickly degraded in plasma and liver. Thus, it has been suggested that systemic CPFO administration does not reach the organs, and that the CPFO found in them has been metabolized from the CPF that reaches the organs [19,20]. Accordingly, we chose CPF as the study compound since it is also the form to which animals and humans are exposed, which would be transformed in the tissues, producing the effects most likely induced by locally metabolized CPF/CPFO.

We chose cholinergic murine neuronal SN56 cells, which are originated from septum BF, to determine the cell death mechanisms that are induced following CPF treatment on BF cholinergic neurons [5]. Cells were cultured following the protocol by Del Pino et al. [5]. CPF has been indirectly shown to be metabolized into CPFO in the SN56 cell line, since acetylcholinesterase (AChE) cannot be inhibited by CPF on its own [21], but this inhibition has been observed in the SN56 cell line following CPF treatment [5]. The dose (0.1 mg/kg) described to inhibit AChE in humans [22] has been estimated to be a concentration of 0.3 µM in brain tissue [23]. We chose a concentration range of 0.1–50 µM, since these concentrations have been shown to inhibit AChE and induce cell death in SN56 cells [5]. In addition, a 30 µM concentration was chosen to test our hypothesis since it was shown to be the minimum concentration that induces P75^NTR^ overexpression and cell death following a single exposure [8].

### 2.2. Analysis of Cell Viability and Caspases Activation

The cell viability of SN56 cells was studied by performing an MTT assay, following the different treatments for one or fourteen days [5]. Apoptotic cell death was detected in SN56 cells using a Caspase-Glo 3/7 luminescence assay kit (Promega, Madrid, Spain), following the producer’s guidelines.

### 2.3. GSK-3β Activity Analysis

The protocol by Moyano et al. [8] was followed to analyze immunoprecipitated GSK-3β in the lysate cells, with the use of a mixture of anti-GSK-3β antibody and agarose beads (EZview Red Protein G Affinity Gel, P6486; Sigma, Madrid, Spain). GSK-3β activity was analyzed using a GSK-3β Activity Assay Kit (CS0990; Sigma, Madrid, Spain), according to the producer’s procedures.

### 2.4. Gene Expression and Protein Content Analysis

Gene expression analysis was performed employing validated primers (SA Biosciences) for mRNAs encoding *P75^NTR^* (PPM04327F), *P38α* (PPM03578A), and *ACTB* (PPM02945B), according to Del Pino et al. [5]. QPCR data were analyzed following the Ct (cycle threshold) method [24].

Quantification of Phospho-p38α (Thr180; p-P38α), p-GSK3β (Ser9), plasminogen activator inhibitor 1 (PAI-1), β-Catenin, Cyclin D1, c-Myc, TrkA, P75^NTR^, proNGF, mNGF, and t-PA protein content was analyzed with commercial ELISA kits (MBS9501531, MBS9501465, MBS261751, MBS724736, MBS9312804, MBS7725905, MBS2022659, MBS2703911, MBS761054, MBS269783, and MBS2511697, respectively, MyBioSource, San Diego, CA, USA), following the producer’s guideline.

### 2.5. Gene Knockdown

SN56 cells were transfected using siRNA (Qiagen, Barcelona, Spain) homologous to mouse *P75^NTR^* (GS18053), and *P38α* (GS26416) target genes following the HiPerfect Transfection reagent guideline. As a transfection control, an All-Stars-Negative-Control siRNA (Qiagen, Barcelona, Spain) was used. The transfection efficiency was measured by performing a gene expression analysis of silenced genes (*P75^NTR^* and *P38α*).

### 2.6. Statistical Analysis

Results from each experimental condition represent the replicates performed at least 3 different times (*n* = 9) and are presented as mean ± standard error of the mean (SEM). Statistical comparisons between control and treatment groups were performed by 1-way ANOVA (different treatment analyses) or 2-way ANOVA (gene manipulation vs treatment analysis). A Tukey post hoc analysis was performed following the analysis of variance. GraphPad software 5.1 was used to perform statistical analysis. Statistical difference was accepted when *p* ≤ 0.05.

## 3. Results and Discussion

**Figure 1:**
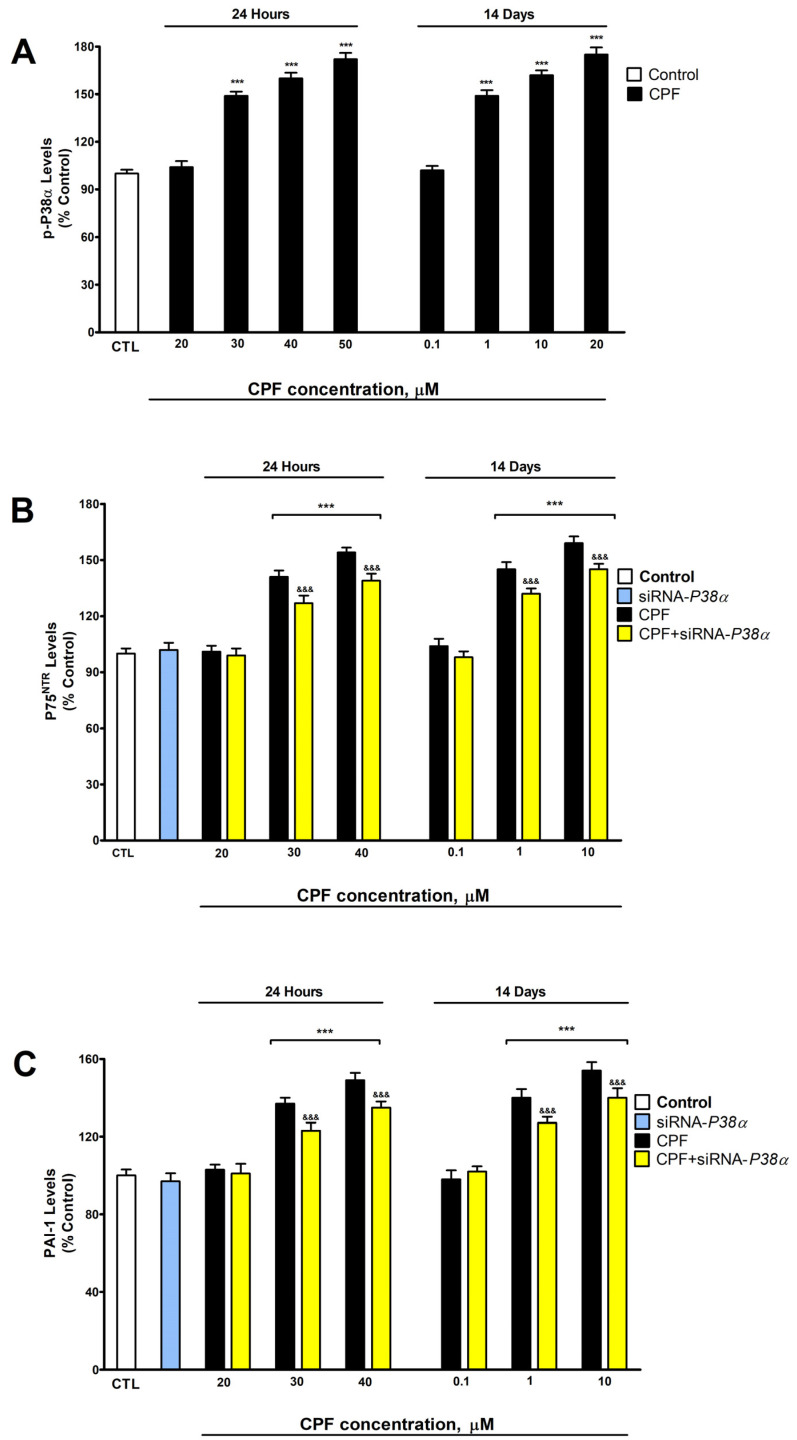
Analysis of CPF action on p-P38α (**A**), P75^NTR^ (**B**), and PAI-1 (**C**) levels. Results are shown as a percentage of control values. Results of protein content were normalized by total protein concentrations. *** *p* ≤ 0.001 compared to control. ^&&&^ *p* ≤ 0.001 compared to *P38α*-silenced cells exposed to CPF.

**Figure 2:**
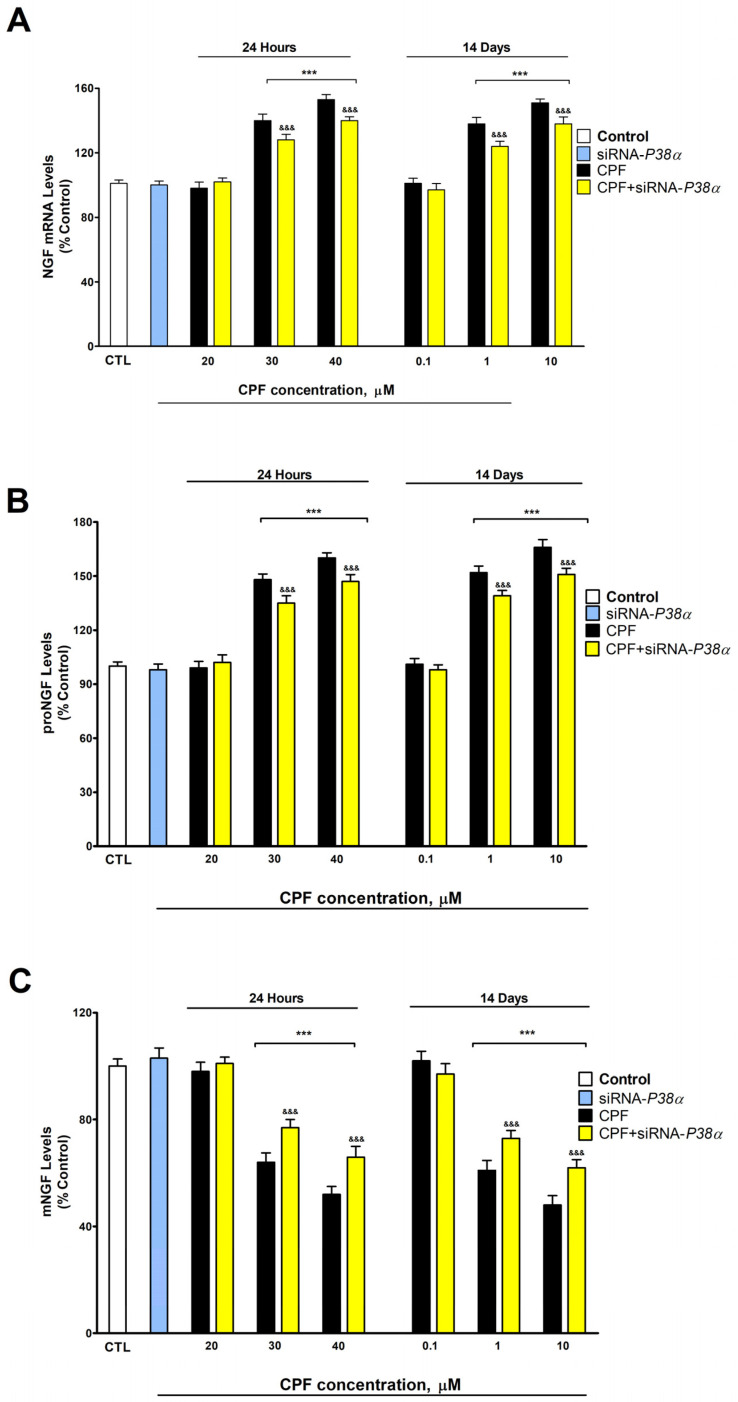
Results from NGF gene expressions (**A**), proNGF (**B**), and mNGF (**C**) levels following one or fourteen days of CPF exposure. Results are shown as a percentage of control values. Results of protein content were normalized by total protein concentrations. *** *p* ≤ 0.001 compared to control. ^&&&^ *p* ≤ 0.001 compared to *P38α*-silenced cells exposed to CPF.

This research displays that CPF increased *Ngf* gene expression and p-P38α, PAI-1, P75^NTR^, and proNGF protein levels, and decreased mNGF protein content following single (from 30 μM) and repeated exposure (from 1 μM) (Figure 1 and Figure 2). Previous studies have shown that CPF upregulates P75^NTR^ in BF SN56 cells [7] and p-P38α in SH-SY5Y cells [14], overexpresses PAI-1 in mouse ileum and colon [25], does not alter TrkA protein content in BF [23], and decreases *Ngf* gene expression during development in rat forebrain [26], which supports our findings. According to our knowledge, the effect of CPF on t-PA, proNGF, and mNGF has not been previously studied. *Ngf* upregulation and PAI-1 protein level increase, which is a tPA inhibitor that inhibits proNGF processing to mNGF [27], probably mediate the decrease in mNGF and the increase in proNGF protein levels produced. However, we cannot discard that other convertases could be altered and contribute to the effect observed.

*P38α* knockdown in CPF-treated cells induced lower upregulation of NGF gene expression, a lower increase in P75*^NTR^*, proNGF, and PAI-1 content, and a lower decrease in mNGF content than that observed in CPF wild-type treated cells, but the reversion was not complete, suggesting that other mechanisms are involved (Figure 1 and Figure 2). p-P38α effects on proNGF and mNGF have not been previously studied. P38α has been reported to upregulate P75*^NTR^* in prostate cancer cell lines [17], PAI-1 in HepG2 cells [28], and NGF gene expression in periodontal ligament-derived fibroblasts [18], supporting our results. CPF overexpresses histone deacetylase 1 (HDAC1) in human breast cancer cells [8], and another member of the family, HDAC2, was reported to increase NGF expression and proNGF and P75^NTR^ protein content, and decrease mNGF protein content in BF SN56 cholinergic cells [12]. Thus, the probable HDAC2 overexpression induced by CPF may also contribute to the disruption of the NGF/P75^NTR^/TrKA signaling pathway.

*P38α* and P75^NTR^ silencing efficiency was evaluated, and negative controls with scrambled siRNA were used to ensure that the effects observed on *P38α* and P75^NTR^ gene expression were produced specifically by the siRNA used to silence these genes (Supplementary Figure S1).

**Figure 3:**
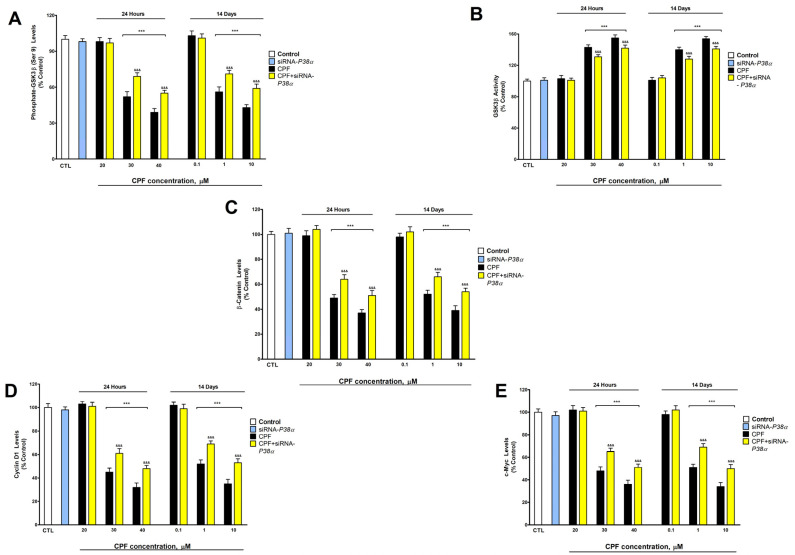
Results from p-GSK3β (Ser9) (**A**) levels, GSK3β activity (**B**), and β-Catenin (**C**), Cyclin D1 (**D**), and c-Myc (**E**) levels following one or fourteen days of CPF exposure. Results are shown as a percentage of control values. Results of protein content were normalized by total protein concentrations. *** *p* ≤ 0.001 compared to control. ^&&&^ *p* ≤ 0.001 compared to *P38α*-silenced cells exposed to CPF.

Moreover, GSK3β activity was increased, but p-GSK3β (Ser9), Cyclin D1, β-Catenin, and c-Myc content was decreased, in a concentration-dependent way, in SN56 cells after single (following 30 µM) and two week (following 1 µM) treatment with CPF (Figure 3). CPF was described to induce the β-Catenin pathway and decrease GSK3β activity at low concentrations when cell proliferation was induced in human breast cancer cells, but produced the opposite effects at higher concentrations, when cell death was observed in these cells, which supports our findings [8]. The decrease in p-GSK3β (Ser9) increases the active enzymatic units, which induces β-Catenin phosphorylation and its degradation, triggering the downregulation of c-Myc, Cyclin D1, and other downstream target genes of the WNT/β-Catenin pathway [29]. Thus, CPF induction of GSK3β activity could mediate the consequent Cyclin D1, β-Catenin, and c-Myc protein level decrease. The silencing of *P38α* partially reverted the WNT/β-Catenin pathway downregulation, indicating that additional mechanisms might also be related to this effect (Figure 1). No previous studies have described WNT/β-Catenin pathway disruption through increased p-P38α content. Activation of P38α has been reported to up- or downregulate the WNT/β-Catenin pathway depending on the type of cells [16,30], supporting our findings. HDAC2 was reported to regulate the WNT/β-Catenin pathway in BFCNs exposed to bisphenol A [29], so this mechanism could also contribute to these alterations. Additionally, the NGF/P75^NTR^/TrkA signaling pathway has been related to the induction of the WNT/β-Catenin pathway by acting on GSK3β [31], and decreased NGF levels induce GSK3β activity [32], as we observed. Thus, this mechanism could also contribute to the WNT/β-Catenin pathway downregulation shown.

**Figure 4:**
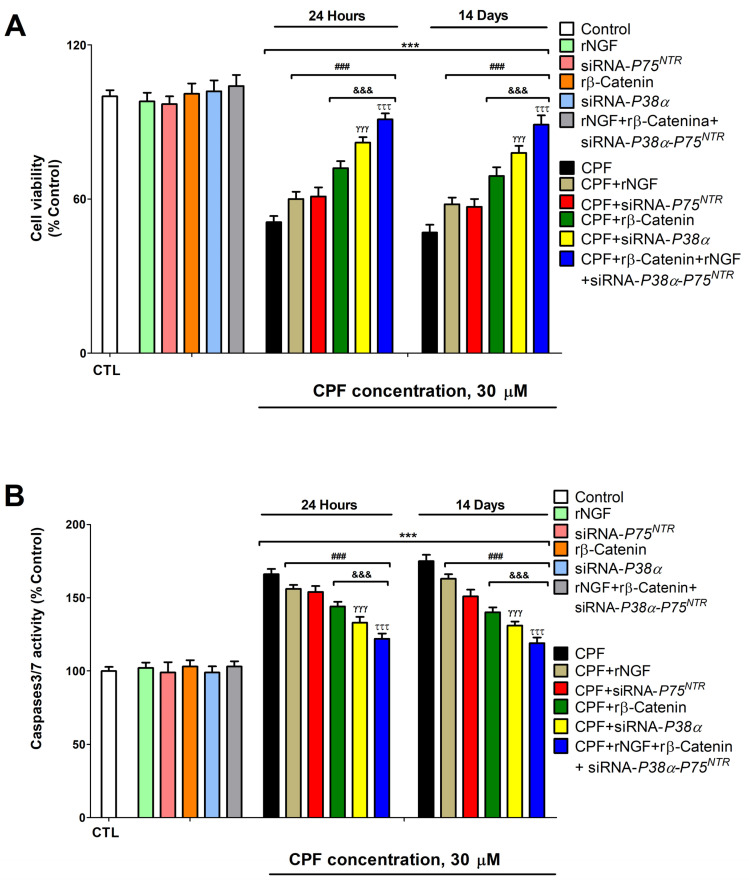
Results from cell viability (MTT assay) (**A**) and apoptosis (caspase 3/7 activity assay) (**B**). Results were normalized by total protein concentrations. Results are shown as a percentage of control values. *** *p* ≤ 0.001 compared to control. ^###^ *p* ≤ 0.001 compared to CPF treatment. ^&&&^ *p* ≤ 0.001 compared to *P75^NTR^*-silenced cells treated with CPF. ^γγγ^ *p* ≤ 0.001 compared to rβ-Catenin and CPF treatment. ^τττ^ *p* ≤ 0.001 compared to *P38α*-silenced cells treated with CPF.

Ultimately, CPF (1 day and 2 week) treatment induced SN56 cell viability reduction (MTT assay) and apoptotic cell death (caspases 3/7 assays) (Figure 4), as previously reported [5]. This reduction in viability and increase in apoptotic cell death was higher after 14 days of treatment. Although the difference in apoptotic cell death was significant, it was a small one, which could be related to the fact that at low concentrations of CPF, the repeated treatment did not induce a great effect. A partial reversion of these results was observed in SN56 cells silenced against *P38α* or *P75^NTR^* treated with CPF, in SN56 wild-type cells treated with CPF and rβ-catenin or rNGF, and in SN56 cells simultaneously silenced against *P38α* and *P75^NTR^* treated with rβ-Catenin, rNGF, and CPF, which shows the involvement of these mechanisms in the cell death observed following CPF treatment of wild-type cells. P75^NTR^ overexpression in BF cholinergic SN56 cells treated (single and repeated) with CPF mediates cell death induction [8]. P38α upregulation was reported to induce BFCN death, and its inhibition blocks it [15]. P75^NTR^ receptors are activated by proNGF, triggering BFCN apoptotic cell death [12]. On the contrary, the TrkA receptor is activated by mNGF, which keeps BFCNs alive [12]. ProNGF increase or NGF reduction induced cell death [12,13]. All of the previous studies support our findings.

However, additional mechanisms seem to be involved in the apoptotic cell death observed. In this regard, HDAC2 has been reported to induce BF SN56 cell death [12,29]. CPF upregulates the AChE S variant, downregulates alpha-7 nicotinic receptors, and induces oxidative stress, leading to BF SN56 cell death [7]. Thus, these effects may also contribute to the cell death observed in BF SN56 cells induced after CPF treatment.

## 4. Conclusions

To summarize, CPF (1 day and 2 weeks of exposure) increased p-P38α levels, downregulating the NGF/P75^NTR^/TrkA pathway and WNT/β-Catenin pathway, through increased GSK3β activity, leading to BF cholinergic SN56 neuron death. Additional studies should be developed to uncover the mechanisms involved in NGF/P75^NTR^/TrkA and WNT/β-Catenin pathways disruption and the triggering of cell death and to confirm in vivo their involvement in cognitive decline. The findings shown in this work may help to reveal the pathway through which CPF triggers cognition decline and provide additional tools to prevent and manage these processes.

## Data Availability

The original contributions presented in the study are included in the article/Supplementary Material, further inquiries can be directed to the corresponding authors.

## Supplementary Materials

The following supporting information can be downloaded at: https://www.mdpi.com/article/10.3390/foods13152427/s1, Figure S1: (**A**) *P38α* and *P75^NTR^* mRNA levels after single silencing. (**B**) *P38α* and *P75^NTR^* mRNA levels after concomitant silencing. Data represent the mean ± SEM of three separate experiments from cells of different cultures, each performed in triplicate. *** *p* ≤ 0.001, significantly different from controls.
