# Supplementary material for: Increased Levels of Phosphorylated-P38α Induce WNT/β-Catenin and NGF/P75NTR/TrkA Pathways Disruption and SN56 Cell Death following Single and Repeated Chlorpyrifos Treatment
Source: Foods. 2024 Aug 1;13(15):2427. doi: 10.3390/foods13152427 (PMC11311586; doi:10.3390/foods13152427)
Supplement: Supplementary file 1 [file foods-13-02427-s001.zip › foods-3077562-supplementary.pdf]

Figure Supplementary Legend.

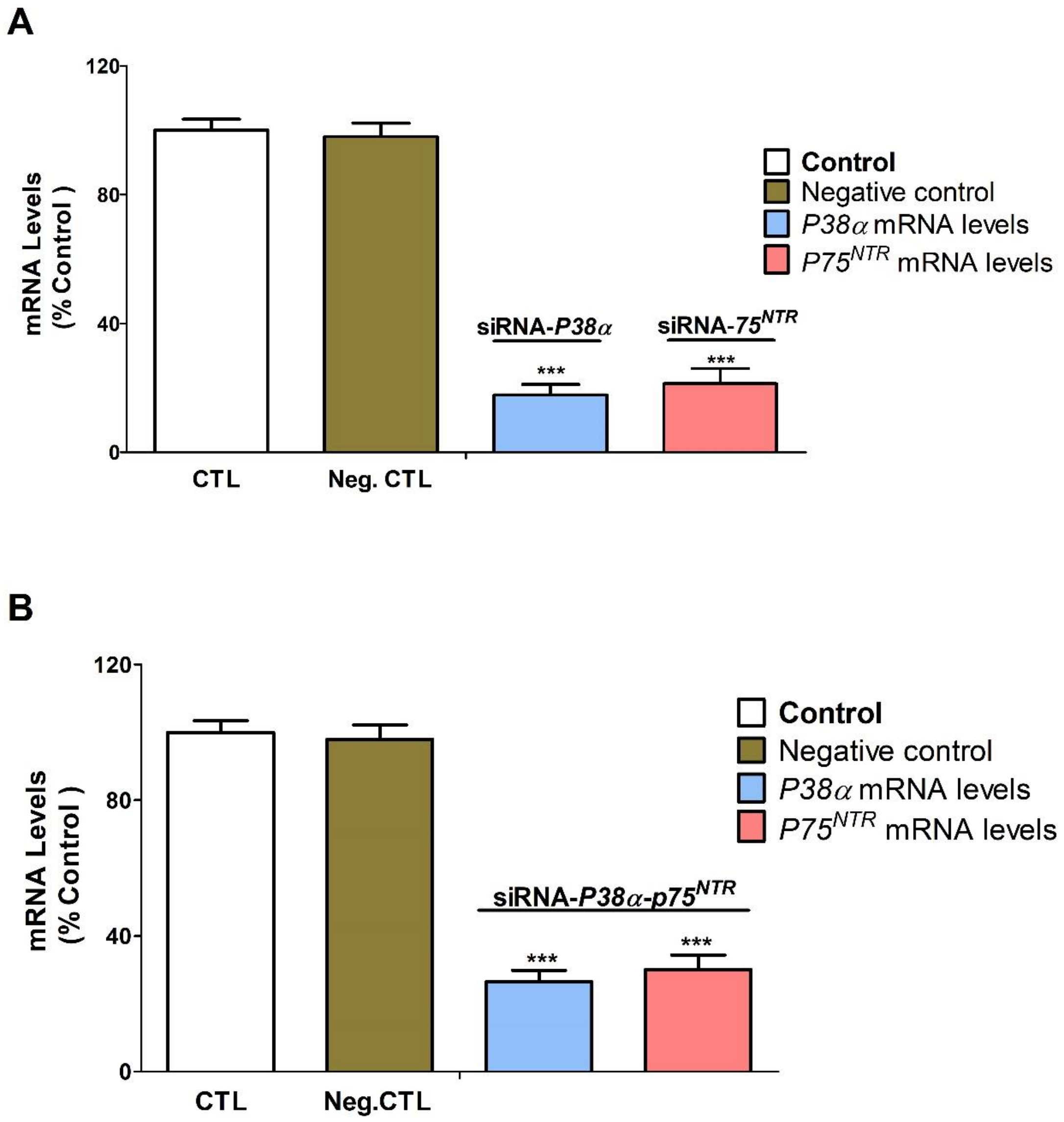

Supplementary Figure S1. (A) *P38α* and *P75*^{NTR} mRNA levels after single silencing. (B) *P38α* and *P75*^{NTR} mRNA levels after concomitant silencing. Data represent the mean ± SEM of three separate experiments from cells of different cultures, each performed in triplicate. $^{***}p \leq 0.001$, significantly different from controls.
